# Ethics, design, and implementation criteria of digital assistive technologies for people with dementia from a multiple stakeholder perspective: a qualitative study

**DOI:** 10.1186/s12910-024-01080-6

**Published:** 2024-07-27

**Authors:** Stefanie Köhler, Julia Perry, Olga A. Biernetzky, Thomas Kirste, Stefan J. Teipel

**Affiliations:** 1 Deutsches Zentrum für Neurodegenerative Erkrankungen, Site Rostock/Greifswald, Gehlsheimer Str. 20, Rostock, 18147 Germany; 2 Section for Gerontopsychosomatic and Dementia Diseases, University Medical Center Rostock, Rostock, Germany; 3 Faculty of Informatics and Electrical Engineering, Institute for Visual & Analytic Computing, Faculty of Informatics and Electrical Engineering, University of Rostock, Rostock, Germany; 4 Department of Medical Ethics and History of Medicine, University Medical Center Göttingen, Göttingen, Germany

**Keywords:** Dementia, Assistive technology, Ethics, Caregiving

## Abstract

**Background:**

Dementia impairs the ability of people with dementia to be autonomous and independent. They need support from third parties, who should ideally respect their autonomy and independence as much as possible. Supporting people with dementia can be very burdensome for caregivers and numbers of patients increase while numbers of potential caregivers decline. Digital assistive technologies (DATs) that directly support patients or their caregivers may help bridging the increasing gap between need of support and available resources. DATs have the potential to preserve the autonomy and independence of people with dementia and promote their abilities, if they are properly designed in close interaction with future users. In our study, we focused on ethical concerns, technological requirements, and implementation criteria for DAT in general and specifically to support outdoor mobility of people with dementia.

**Methods:**

We applied a qualitative approach and conducted a World Café (2 tables, *n* = 7) and an online focus group (*n* = 6) with people with dementia, relatives, healthcare professionals, scientists, ethics experts, and experts for digitally-assisted medical care. We descriptively analyzed the data using a content analysis approach.

**Results:**

The participants reported technological (e.g., lack of Wi-Fi), financial (e.g., expensive devices or lack of budget for DATs), political (e.g., legal hurdles such as the European Medical Device Law or data protection regulations) as well as user-related hurdles (e.g., lack of digital competence) for the implementation of DAT in dementia care. Among the issues discussed were the importance of autonomy, independence, safety, privacy, and questions of decision making capacity in DAT’s use. Participants identified opportunities and benefits in self-learning, situation-aware DATs and wished for dementia-friendly communities. They emphasized the value of personal interaction that should not be replaced, but rather supported by DAT.

**Conclusion:**

The results revealed multiple hurdles and ethical concerns for DAT use and provided recommendations for designing and implementing DATs. Further investigations are needed on the impact of DAT on personal interactions in caregiving and the role of DAT in dementia-friendly communities.

**Supplementary Information:**

The online version contains supplementary material available at 10.1186/s12910-024-01080-6.

## Introduction

Caring for people with dementia is not only emotionally and physically challenging, but also time consuming [1–4]. Worldwide, family caregivers of people with dementia spent about five hours a day providing domestic care [5]. People with dementia rely more often on institutional care compared to people of similar age who do not have dementia [6, 7], but shortage of medical staff increasingly hampers high quality and dementia-specific care [2, 8, 9]. Despite their need of care, people with dementia wish to maintain their independence and autonomy [10–12]. The dementia-related increase in dependence on support from others [9] stands in opposition to the wish for autonomy [12]. Coping with everyday life, such as shopping, visiting friends or a doctor, and participating in sports or cultural activities, is very important for the autonomy of people with dementia [11]. Furthermore, mobility is crucial for identity, well-being, and social connectedness [13]. As people with dementia often experience disorientation even in the early stage of the disease [14], the lived space decreases [13] while getting-lost events increase [15]. Furthermore, people with dementia reported to feel vulnerable and embarrassed in public spaces [13].

Digital assistive technology (DAT), such as smart home technologies, robots, smartphones e.g., with navigation app and GPS tracking, or digital games [16] could relieve formal and family caregivers and support people with dementia to maintain their autonomy and independence in general and mobility in particular [17]. We provided a definition of assistive technology (AT) in Text Box 1.

**Table:** 

Text Box 1: Definition of Assistive Technologies and Ambient Assisted Living
The World Health Organization's (WHO's) Global Cooperation on Assistive Technology (GATE) defined assistive technology (AT) as “(..) the application of organized knowledge and skills related to assistive products, including systems and services“ [18]. The WHO described “assistive products as devices, equipment, instruments or software from 6 functional domains: mobility, vision, hearing, communication, cognition and self-care. Examples of assistive products are physical products such as wheelchairs, spectacles and hearing aids, and digital products such as software and apps.” [19]. In our study, we focused on AT in the sense of digital assistive technologies. Ambient Assisted Living (AAL) encompasses the interaction of digital devices and includes activity recognition or the Internet of Things [20]. AAL aims to support people to live in their familiar environment by enhancing their independence, mobility, self-confidence, and autonomy by compensating or preventing cognitive or physical disabilities [21]. Hereby, social connectivity is an important part of AAL to prevent social isolation, increase safety, and involve healthcare providers and family caregivers in the care of (older) people with declining health [21]. Therefore, AT could play a crucial role in the aging-in-place paradigm, which allows people with dementia to live at home for longer rather than in institutional care. Although people with dementia often are the primary and direct users of DATs, they are often ignored in the development of AAL technologies and replaced by the involvement of secondary users such as relatives or healthcare professionals [20].

Technological support of people with dementia requires that DAT respects and supports the primary users’ needs and requirements [11, 22]. People with dementia and caregiving relatives perceived different values to be important for the use of DAT [23] which motivates the inclusion of people with dementia and their stakeholders into the design of DATs.

**Fig. 1:**
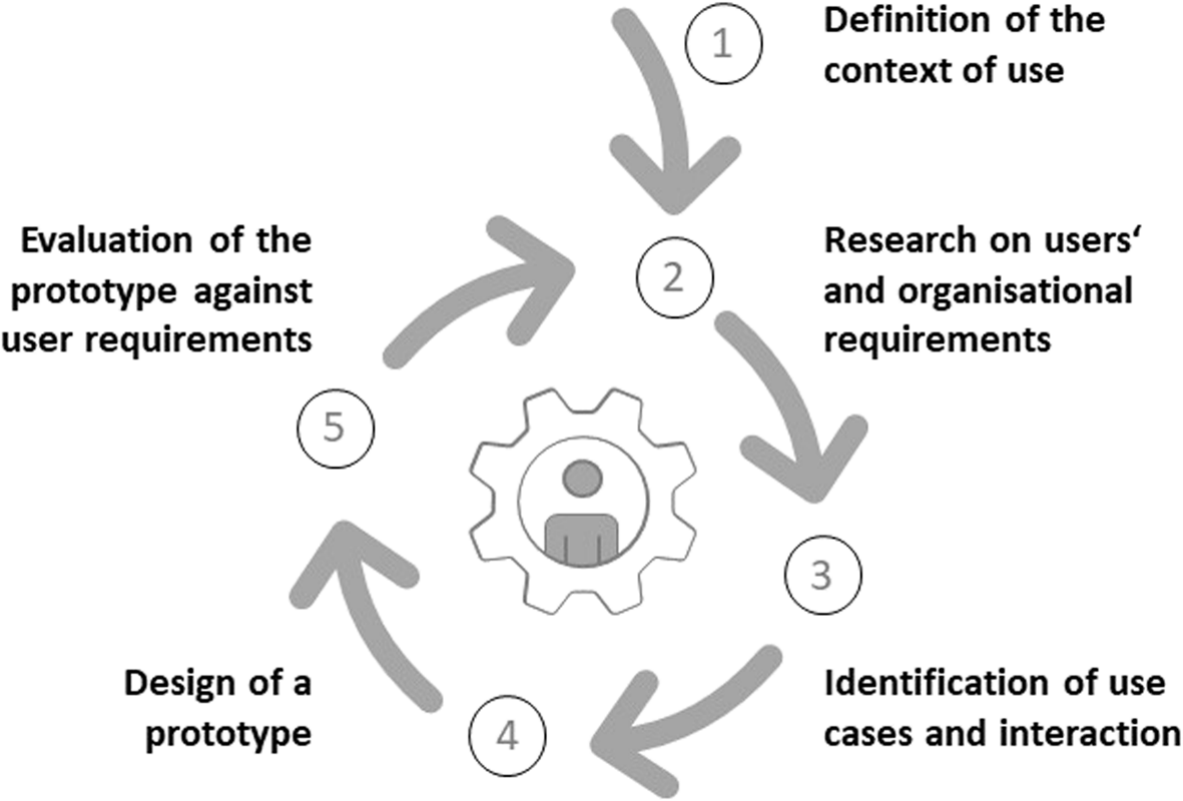
User-centered design process (based on Bevan, [25] and Lubis et al., [26])

The user-centered design (UCD) enables to capture the needs and values of different users. UCD integrates the future users in designing DAT to enhance acceptance and usage of DAT [24]. The UCD process (see Fig. 1) includes identifying the context in which the technology will be used, researching users’ and organizational requirements, defining of use cases and the interaction between user and device, designing a prototype, and evaluating the prototype with future users [25, 26].

Besides technological requirements, such as design and functions, DAT should incorporate users’ values [27] and undergo ethical reflections, especially in the context of medical care for people with dementia [28–30]. However, Diaz-Orueta, Hopper, and Konstantinidis [31] identified a lack of ethical consideration of researchers in working with people with dementia in the design process of DAT. Ethical recommendation for the use of AT exist e.g., from the Alzheimer’s Societies of Canada and UK, from the Nuffield Council on Bioethics, and from the EU-funded research and development technology project “Assisting family Carers through the use of Telematics Interventions to meet Older persons’ Needs (ACTION)” [32]. Still, the majority of AT (67%) have been developed without paying attention to ethical considerations [33]. The gap between theoretical discussion and actual involvement in technology development may indicate a greater need for interdisciplinary research projects to develop AT for people with dementia. The research questions developed for this study are based on our previous work [11]. We had investigated the affinity for technology and the needs of people with dementia regarding DAT to support outdoor mobility. In addition to needs and requirements for DAT, the study also revealed differences in mobility of people with dementia between rural and urban areas. Here, we present the discussion on DAT from a broader perspective due to the inclusion of dementia-, healthcare-, IT-, and ethics-experts following the first three steps of the UCD process.

## Objectives

Our study explored the preferences and concerns of people with dementia and other stakeholders about the use and application of DAT in dementia care within a multidisciplinary approach. Additionally, we aimed to identify the participants’ views on risks and chances associated with DAT. Participants discussed the following questions:

1. Which needs should DAT address to support people with dementia and healthcare in dementia?
2. Which application fields, needs, and benefits exist regarding DAT supporting mobility? What are limitations and hurdles?
3. How can DAT support outdoor mobility of people with dementia?
4. Which differences exist regarding outdoor mobility needs between urban and rural areas? Which chances and risks exist? Which general conditions must be fulfilled?

## Methods

### Recruitment

We recruited participants from previous research projects such as TaNDeM - German network for translational dementia care research [34], previous investigations as part of the EIDEC project-Ethical and Social Issues of Co-intelligent Sensory Dementia Care (EIDEC), and from the German Alzheimer’s Association. Participants working at a university were recruited from the researchers’ network. Our inclusion criteria included experts in dementia care, support, or research, either experts by experience or experts by profession. We invited in total 18 persons to join the World Café: persons from the regional Alzheimer’s Association, experts in digital health applications, caregiving relatives of people with dementia, persons working in a nursing home, members of the State Senior Citizen Advisory Board (two each), three professors of health/nursing science or medical ethics, five scientists in dementia research or nursing science. Furthermore, we asked nine persons for participation in our online focus group: one person from the regional Alzheimer’s Association, one employee of an institution for dementia support, one representative of a health insurance company, one caregiving relative, one scientist in dementia research, one consultant in the department of ageing, care and disability, one manager of a nursing home, and two people with dementia. We informed participants about the opportunity to participate by telephone or email. Participants rejected participation due to a lack of time (8) or acute illness (3). Three candidates did not respond to the invitation. In total, 13 participants joined the discussions (see Table 1). We planned the discussions for two different appointments with different stakeholders. We held one in-person discussion as a World Café and one focus group as a zoom online meeting. Our goal was to ensure diversity in terms of sex and (occupational) background when determining the composition of each group.

**Table 1:** Characterization of participants

Group (*n* = 13)	Sex	Background/Occupation
World Café table 1 (*n* = 4)	female	Expert in healthcare, Member of a regional Alzheimer’s Association
	female	Quality manager at a nursing home
	male	Expert in ethics
	male	Member of the State Senior Citizen Advisory Board
World Café table 2 (*n* = 3)	female	Nursing manager at a nursing home
	female	Senior Strategy Manager for digitally-assisted medical care
	male	Male nurse and scientist for nursing science
Online focus group (*n* = 6)	female	Spouse of a person with dementia
	female	Employee in an institution for dementia support
	female	Scientist in dementia research
	male	Person with dementia
	male	Person with dementia, Member of an advisory board of people with dementia
	male	Manager of a nursing home

### Procedure

Focus group discussions such as World Cafés or online discussions can give an overview about relevant themes from different perspectives [35]. The interaction and discussion between participants improves data quality [35]. Furthermore, focus group discussions enable to collect the perspective of many people in a time-effective way [35]. Therefore, we decided to implement these method in our study.

#### World Café

During the World Café, participants discussed questions 1 and 2 on needs, application fields, and benefits regarding DAT in healthcare and DAT in supporting mobility. We prepared two different tables with a tablecloth made from flipchart paper. The questions to be discussed were visualized on the paper tablecloth to ease understanding and staying on topic. Furthermore, we invited our participants to independently document their ideas and arguments on paper. Two project members kept one protocol per table to document the results. Each table took 30 to 40 min for discussion and was moderated by one female researcher (O.A.B. & S.K.) with experience in participatory research. The moderators structured the discussion into the following points: greeting, introduction of the participants, presenting the question to be discussed, and if possible summarizing the results of the first discussion round. During discussion, moderators asked in-depth questions or clarified the correct understanding of participants’ arguments and ideas. Furthermore, they ensured that everyone had the opportunity to contribute to the discussion. After a short break, participants but not the moderators changed tables.

#### Focus group

Due to the COVID-19-pandemic, we decided to keep the risk of infection for people with dementia low by offering an online focus group interview. In addition, our goal was to deepen and expand the results of the World Café in the online focus group. During the online focus group discussion, we concentrated on outdoor mobility (question 3 and 4) as this had not been discussed sufficiently before. One week before the discussion, we sent the results of the World Café to the participants of the online focus group. Providing the results in advance enabled participants to get used to the previous results and promoted in-depth discussion. Therefore, we summarized the results of the World Café in an anonymized and easy to understand way. We planned 60 min for the online-meeting including 30 min for discussion. We structured the online focus group interview as follows: greeting, introduction of the participants, warm-up-question, discussion, summary of the discussion, and acknowledgement. Two project members wrote a protocol of the discussion. After obtaining consent, a video call recording completed the log. The moderator (S.K.) from the World Café led the focus group discussion.

### Analysis

After discussions, participants and project members received the structured protocols. We asked them to validate the protocols and to give feedback if they had additions or disagreed with the content. They got two weeks to provide their feedback. In total, one participant and three project members gave feedback. One participant asked for deleting their quotations and for paraphrasing their comments. One participant of the online focus group communicated their interest in the topic after receiving the structured protocols for preparation. We analyzed, structured, and visualized the data from the protocols using a content analysis [35]. Content analysis enables a reduction of the volume of qualitative data by identifying patterns and themes in the material [35]. Patterns can be seen as umbrella term of categories or topics [35]. In our study, we inductively built patterns from paragraphs, word groups, or sentences in the protocols. After generating patterns, we deductively sorted themes to matching patterns. Themes describe the patterns in more detail and give context [35]. For example, the sentence: “Our participants worry when their loved ones go outside alone in the dark.” leads to the theme “going out alone in the dark” which belongs to the pattern “concerns”. Here, we combined all responses from participants related to this pattern.

## Results

We identified nine themes from the material: ethical values and DAT; acceptance of DAT; concerns regarding DAT; technological, administrative, and political hurdles in the usage of DAT; desires regarding DAT; user-related hurdles in the usage of DAT; use cases for DAT; facilitators for outdoor mobility; and differences in mobility and DAT between urban and rural areas. As only the online focus group discussion was recorded, we only cite quotations from the online focus group participants.

### Ethical values and DAT

Participants discussed how the use of DAT impacts the values of autonomy, safety, social interaction, and privacy. They desired DAT that promoted autonomy and safety, associating autonomy with the independent management of daily life. Safety was mainly associated with physical integrity which is threatened by wandering tendencies, falls, and the inability to live at home. Participants feared a reduced autonomy if DATs took too many tasks away from people with dementia, resulting in a more rapid increase of cognitive impairment and dependence. They highlighted the need for a situation-aware assistance which only supports in cases of need and promotes abilities and activities. Situation-aware DAT should be ability- instead of deficit-oriented. However, users should be able to refuse DAT’s support to prioritize autonomy over safety. In case of refusal of the support of DAT, participants raised the question as to who would turn off the DAT: The user with dementia or the DAT itself?

Participants further asked for whom safety should be provided by DAT: people with dementia or caregivers? One participant saw difficulties in using DAT to impede wandering tendencies as locating and door locking systems constitute deprivation of liberty and require a judicial decision. Further, participants raised the question of privacy and personal boundaries: Should DATs such as robots be programmed to touch people and if yes, how often, in which context, and on which part of the body?

DAT should support caregivers and “save time, which can be used for personal interaction”. Participants rejected the idea that DAT could substitute personal interaction. Participants emphasized that personally accompanying people with dementia to an appointment brings more ethical value than just ensuring that they arrive at the right place at the right time. Accompanying a person has a social and a medical value as caregivers can talk to the person in need and evaluate their health condition. Therefore, they agreed to the use of DAT only if it saves time for additional personal interaction with the person in need.

For choosing a DAT for people with dementia, decision making strategies as nudging should be avoided as this would lead to an ethical dilemma. They defined nudging as discrete manipulation in decision-making such as positioning healthy food in the range of people’s vision. Our participants feared manipulation and violation of autonomy and free choice of people with dementia due to nudging.

### Acceptance of DAT

Participants of the World Café suggested to include people with dementia, caregiving relatives, and healthcare professionals in the development of DAT to increase the benefits and acceptance of DAT. For implementation of DAT, they also wished for integrating (nursing home) managers, IT managers of the institution, physical and occupational therapists, physicians, and product designers of digital devices. They suggested a collaboration among all professional groups from the beginning as prerequisite for a successful and need-oriented implementation of DAT. Participants of the online focus group preferred DAT as a common device which adapts automatically to the needs and resources of people with dementia:

“Well, what I want to say is: there’s a bunch of stuff, the only problem is that those affected have different stages of the disease (…), but if the system is trained, then it works.” (Participant with dementia from the online focus group).

Gamification elements and incentives such as a massage, purring of an animal robot, or a visual feedback (e.g., interactive therapy balls) should be used to maintain motivation and acceptance and to leverage the reduced concentration span of people with dementia. At the same time, participants warned of technology being too human-like because people could be scared by that similarity. Participants felt that humanizing robots was problematic because people with dementia would not be able to distinguish between humans and robots.

People with dementia should get familiar with DATs in an early stage of their impairment to ensure DATs’ acceptance in a later stage. To ensure acceptance, participants demanded an early analysis of users’ needs within a home-based screening. The screening should take the diversity of dementia symptoms into account. In addition, DATs should take gender-specific preferences into account. The voice output of DAT should integrate female as well as male voices to allow for user preferences.

Participants perceived a higher acceptance of medical aids such as walkers in a nursing home than in public because many patients use medical aids which leads to a kind of group membership. The personal and public view of medical aids should evolve from “medical devices to status symbols”. DATs would have the potential to be life-style-products which can be used confidently instead of stressing need for help.

Participants mentioned that dementia-specific DATs are needful and user-friendly, but also “insanely expensive” (participant from the online focus group). Further, they recommended adapting ordinary existing devices to the need of people with dementia instead of developing expensive specialized devices. Features, such as fall detection and sensors to measure vital signs, would enhance the benefit and implementation of DATs in institutional care. A connection to the institutional network and access to Wi-Fi or Bluetooth should realize data transmission.

Participants pointed out that apps and smart home systems are easily accessible, but would miss the reality of life of people with dementia:

“All that mobile phone and smartwatch stuff. That’s common practice. Anyone can buy that. For a few euros, ten or a hundred. (…) I’ve just noticed (…) that many of those affected now can’t do it for themselves. They don’t understand that at all.” (participant with dementia from the online focus group).

Missing the reality of people with dementia would lead to non-use or would overwhelm users.

Participants summarized that DAT should follow a holistic and need-oriented approach to be accepted and useful. DATs should support each person integrated in healthcare and provide the optimal benefit.

### Concerns regarding DAT

Three participants worried about the use of robots in dementia care. They emphasized the importance of emotions in dealing and communicating with people with dementia which robots could not express or react on adequately. Participants noted that especially robots are perceived as strange or repulsive and are seen mostly as a toy than as an assistance.

Participants further expressed concerns regarding hygiene standards. One participant reported to not use the robot seal Paro [36] in her nursing home because its fur cannot be cleaned sufficiently or machine-washed.

### Technological, administrative, and political hurdles in the usage of DAT

Participants discussed the lack of basic technological infrastructure in healthcare. A lack of Wi-Fi would impede the use of cloud-based DAT and online applications. Participants emphasized the importance of interoperability between different DATs, including sensor-based DAT. They named “isolated solutions” as a problem in using DAT and asked for integrated solutions, such as having a scale automatically transfer the patient’s weight into the patient record. For data transmission fulfilling the national data security regulations, data security concepts are missing.

Besides technological hurdles, they named a lack of time to introduce new technologies in their daily work as a hurdle for using DAT.

Participants questioned if the German funding system is able to adopt novel technologies for the benefit of patients and caregivers. Questions of liability in the use of DAT in healthcare remain unclear. Funding of DATs is possible if they have an approval as medical device by law. The approval is expensive and time-consuming due to high administrative hurdles and need of proof of effectiveness. Companies would avoid approval and focus on other application fields which hampers technological innovations in healthcare. Participants criticized the limited budget for medical aids in institutional care and the missing financial resources for purchasing DATs. Participants hoped for facilitated approval of applications and reimbursement of medical devices by healthcare insurances due to changes in legislation such as the new regulation for digital nursing applications.

### Needs regarding DAT

Participants identified a lack of transfer of technologies into healthcare practice. They wished for a better, immediate transfer and implementation of useful DAT into healthcare. A seemingly simple example was an intercom system. This would help prioritizing residents’ needs and saving caregivers’ time. Although such systems have been available for decades, they have rarely been used in nursing homes or other facilities.

Additionally, participants asked for an overview of existing and user-friendly DATs. Designers and scientists should focus on usability testing including the target group of the DAT. Furthermore, research should focus more on existing DATs regarding acceptance and usefulness rather than on developing further DATs which may not be used in the end. They discussed if a central institution is needed to evaluate and collect existing, digital health and care applications and make them available and visible for the public. They preferred a data bank to evaluate and collect applications. Additionally, they spoke out for interoperability between devices starting with uniform charging cables.

### User-related hurdles in the usage of DAT

In general, participants stated a poor digital competence of people with dementia, relatives, and healthcare professionals as a hurdle for the use of DAT. Some people might be overwhelmed by technology. They expected a generational change which will lead to more digital competency in (older) people. Participants identified their lack of knowledge about existing, useful technologies as a hurdle for usage of DAT.

In addition, impairment of hearing (for understanding audio instructions) and eyesight could hinder the use of DAT especially in older people.

### Use cases for DAT

Participants had many different ideas of use cases and designs for DAT. Tablets should provide entertainment for people with dementia such as singing, dancing, and playing. Although participants were concerned about the use of robots, they discussed use cases for robots in dementia care. If robots were used, they should support activities of daily life and reduce or handle aggressive behavior due to emotion recognition. The participants discussed the use of robots in the context of institutional care. In institutional care, personalized, electronic keys with iris scan should help with saving privacy by enabling people with dementia only to enter common or their own rooms.

Furthermore, DAT should help with stabilizing the day-night-rhythm and should provide assistance in case of disorientation. Participants evaluated GPS-trackers in a smartwatch-design as useful to locate people with dementia if they get lost. The smartwatch should navigate people with dementia and support people with dementia in case of disorientation by calling contact persons and transmitting their location. One man with dementia stated a smart home system to be helpful outdoors for navigation and at home for checking the weather and reminding on appointments for instance. Besides navigational aid, one participant named an app as inexpensive substitute for home emergency call systems.

### Support for outdoor mobility

Regarding outdoor mobility, participants agreed that preserving autonomy of people with dementia and keeping “what you have, for example living in a residential area, for as long as possible” (participant from the online focus group) were priorities. Participants spoke out against “dementia-villages” which are designed specifically for people with dementia, but would exclude them from society.

Increasing disorientation in a person with dementia could lead to getting lost and they might become confused by traffic. Therefore, participants wished for involving people from the environment of people with dementia and informing them about the need to support wayfinding: “that you [the baker, S.K.] simply say, here are the rolls and remember, turn right over there” (participant from the online focus group).

Participants named reaching destinations within walking distance, implementing dementia-friendly architecture, e.g. providing urban guidance systems for people with dementia, or a good connection to public transport as supportive for mobility. In case of disorientation, people would prefer to ask passersby for directions or call a relative rather than use a technological device. Multiple persons could act as contact persons to ensure accessibility for people with dementia. Participants emphasized social interaction, especially communication, as essential for outdoor mobility of people with dementia. Rural areas have the advantage of short, familiar, and clear routes. Participants questioned the need of DAT supporting mobility in rural areas:

“The smaller the structures are, in a village where there is only one bakery, it is of course potentially easier for me to find my way around and I may not need the [DAT, S.K.] at all” (participant from the online focus group).

In case of disorientation, participants mentioned that DATs are not part of the coping strategy of people with dementia. Some participants questioned if DAT can support mobility of people with dementia. They felt that social interaction can better promote mobility than a DAT e.g., by accompaniment by relatives and friends, outdoor sports groups, or groups including people with different impairments:

“For example, I go out as a group. So two or three disabled people are walking around outside. They could also help each other.” (participant with dementia from the online focus group).

### Differences in mobility and DAT between urban and rural areas

Participants noted major differences between urban and rural areas in terms of public transport and technological infrastructure. Internet connection and mobile data are often poor in rural areas so that DATs for outdoor mobility would have to fall back on GPS alone. Participants mentioned differences within rural areas and cities itself and recommended an individual consideration of each area or city. One participant with dementia named free Wi-Fi hotspots in the city as a useful guidance system to navigate from hotspot to hotspot. The hotspots’ names would help people to orientate and to navigate through the city.

In rural areas, participants described the use of public transport as difficult because of poor accessibility and outdated schedules. However, older people may not have devices that have a mobile Wi-Fi option which hampers use of public transport. Participants explained that in some villages the bus schedule is not up to date. Without internet access, people would be unable to find out about current departure times. They proposed to identify real-world hurdles of technology use for people with dementia and solve them individually.

## Discussion

In our study, different stakeholders, including people with dementia, their relatives, healthcare professionals, scientists, and IT specialists, discussed ideas of DAT; the needs, benefits as well as the hurdles for implementation of DAT in healthcare practice and support of people with dementia. We focused on DAT in general as well as on DAT to support outdoor mobility of people with dementia. Participants discussed values and preferences affected by the use of DAT, structural hurdles for implementing DAT, and requirements for DAT supporting people with dementia.

### Ethical implications

Both discussion groups, the World Café and the online focus group, emphasized the importance of autonomy for people with dementia and the usefulness of DAT to maintain autonomy. Participants reported conflicts between privacy and safety. A systematic review showed that people are willing to trade privacy for autonomy [37]. Our participants recommended a situation-aware assistance to promote users’ abilities when needed. This would also reduce the negative impact of safety on privacy. A systematic review [38] and a qualitative study [9] emphasized the importance of privacy for people with dementia. Unobtrusive sensors, such as GPS or fall sensors, may enable both promoting safety and saving privacy if they only transmit data in case of need to pre-selected persons. In addition, privacy is also a question of protection of personal and medical data. In particular, sensor data could facilitate the establishment of a long-term, location-independent data recording system that extends beyond the confines of scheduled medical appointments [39]. Therefore, we agree with the call of Schicktanz and Schweda [39] for developing practicable data protection concepts that also ensure the privacy of personal, relational, and topological data. The development of these concepts may require a rethinking of aspects of privacy [30].

With respect to autonomy, participants of the World Café (table 2) raised the question of who should be able to turn off the DAT if a person with dementia refuses it. A previous study showed that people with dementia prioritized the value of autonomy, whereas relatives focused on safety [23]. However, relatives mentioned ‘surveillance’ as crucial, but it was not found in the quotes of people with dementia. In line with this, the scoping review of Sundgren, Stolt, and Suhonen [40] reported that relatives would prefer to coerce people with dementia to use an AT in favor of safety. Our participants from all discussion groups emphasized the importance of autonomy for people with dementia which is in contrast with the findings of Kowe et al. [23] and Sundren et al. [40]. Our participants from the World Café raised this question in regard to decision-making capacity and self-determination. Self-determination is a civil right in the United Nations Charter, Article 1 and 55 [41] and the International Covenant on Civil and Political Rights, Article 1 [42] of the United Nations. Both Alzheimer Europe and Kim et al. have synthesized ethical decision-making approaches to AT use, for example from the Nuffield Council on Bioethics, Bjørneby et al., and the American Speech-Language-Hearing Association flowchart [32, 43]. These approaches focus on a joint discussion of ethical dilemmas with each person effected by the use of AT. However, decision-making capacity is context- and situation-specific and fluctuates with the cognitive abilities of people with dementia. It is impaired at the latest stages of the disease [44]. Therefore, in cases of advanced dementia, decision-making capacity can become fluid and tied to specific situations [44]. For example, in case of disorientation, people with dementia experience anxiety and confusion [45] that may affect their ability to make decisions. Given the fluctuating decision-making capacity and disease progression, advance care planning is recommended [44, 46] even in regard to the use of DAT [47]. Advance care planning could also be applied to the use of DAT and help answering the question when to and who can turn off a DAT, also in moments of fear or confusion. However, advance planning assumes people know about the functionality of DAT and their own future situation [48]. Therefore, care advisors should be trained in specification and functionality of DATs to support decision-making and planning the use of DAT. Additionally, it remains unclear whether people would reject a need-oriented, situation-aware DAT if it is unobtrusive and activates only in the situation of need.

Again focusing on autonomy, our participants were critical of the use of technological door locking systems, as these were seen as methods of physical restraint. According to the German Civil Code (BGB), the use of physical restraints requires sufficient justification and a legal decision by the guardianship court [49].

Another discussion point focused on the concept of using gamification and providing incentives (for a definition of gamification and incentives see Text Box 2) or nudging to influence people’s decision making in using DAT. Incentives are benefits that are independent from the care process, such as promising patients to get their favourite food or sweets [50]. Elements of gamification are e.g., high score lists, social competition, unlockable content, and quests or goals [51]. Following the systematic review on gamification for older adults [51], gamification helps improving health related wellbeing, social interaction, motivation, and engagement. In line with this, a systematic review and meta-analysis [52] found that the use of incentives enhanced the adherence of people with dementia or MCI to exercise interventions. Both gamification and incentives might be useful to increase motivation and adherence in people with dementia and dementia care. Nudging is used in healthcare for instance to improve healthy behaviour (e.g. nutrition scores on food). According to Cohen, nudging in healthcare is a form of libertarian paternalism which facilitates an informed consent, preserving patient’s autonomy [53]. As DATs often are complex and their functions difficult to understand, it remains unclear if informed consent can be obtained from people with dementia and their relatives [54]. In contrast to Cohen, our participants feared manipulation and violation of autonomy and free choice of people with dementia through incentives or nudging. These concerns are understandable as marketing interests, personal hardships, or organizational shortcomings of a care facility may lead to the deployment of such a technology without considering the need and well-being of the person with dementia [55]. Nudging and incentives influence decision-making in subtle ways [50, 55], which could help to increase acceptance of DAT by people with advanced dementia. But, the effectiveness of nudging for people with dementia who may have impaired abilities in decision-making remains doubtful [50]. In conclusion, the challenge of incentives or nudging to encourage the use of DAT lies in preserving free choice and avoiding manipulation. Regarding the use of DAT, nudging and incentives could be seen as soft aid in decision-making if default points have been clarified: Who decides which DAT is best for people with dementia? How can we ensure that the decision to use nudging is made in the patient’s best interest and not for personal or economic reasons? Which incentives are effective and desirable for people with dementia? These questions should be addressed in future studies.

**Table:** 

Text Box 2: Definition of Gamification and Incentive
The Oxford dictionary defines gamification as “the use of elements of game-playing in another activity, usually in order to make that activity more interesting” [56].
An incentive is a reward, an additional benefit which is independent of the primary caring goal e.g., a massage or a pleasing reaction of the DAT (purring, flashing, applause) [50, 57].

### Design requirements and image change

As a result of the World Café discussion, we found that participants were undecided about how the DAT should be designed. In order to make the discussion about DATs more concrete and tangible, we focused on outdoor mobility advices for the online discussion. Here, participants seemed to prefer DATs integrated in familiar and portable devices such as a smartwatch for supporting outdoor mobility. Non-wearable devices like robots should only be applied in institutional care to avoid stigmatization. Our participants stated that a gender specific adaptation of the voice of the DAT to the different users would be helpful. This is in agreement with a previous interview study with 20 participants, ten caregiving relatives, and ten healthcare professionals where a socially assistive robot was tested [58]. Our participants highlighted the need for adapting the DAT to the demands of different target groups (people with dementia, family caregiver, or nurse). Also Wu et al. [58] described different needs of nurses and family caregivers regarding DAT. According to Wu and colleagues [58], family caregivers focused more on social aspects of the robot such as accompaniment whereas healthcare professionals emphasized assistive aspects to relief them from workload. Additionally, our participants from all discussion groups discussed different hardware devices for a DAT. While the World Café participants discussed DAT, such as robots, fall detection mats, intercoms, and belts, the focus group participants focused mainly on wearables, such as smartphones or smartwatches. This need for flexibility and variability of features and design underlines the findings of previous studies arguing for highly customizable, personalized DATs to increase the uptake of DATs in healthcare [11, 58, 59]. As a result of our study, we suggest a modular system of different supporting and monitoring opportunities which can be selected in accordance to the user’s needs. This system should run on different devices and be compatible with the usual operating systems such as Android or IOS.

Our participants felt that the image of assisting or supporting devices should change in the future. Currently, from their perspective, assisting devices indicate the need for help. This result stands in line with a systematic review [60] focusing on acceptability and usability of technology in people with cognitive impairment. The authors identified fear of stigmatization as a reason for people with cognitive impairment to not use assistive technology [60]. The authors suggested devices which reflect the user’s identity [60]. In contrast, our participants preferred a mainstream market approach to an individualistic one. They wished that the image of DAT should change so that they are seen more as a life-style-product than as help for the helpless. This suggestion might be comparable to the use of prescription glasses. Some years ago, prescription glasses were unstylish, pragmatically designed visual aids which transformed to fashion accessories even for people without visual impairment. DAT has the potential to be a medical aid with life-style-product character if design, healthcare, and technology experts design DAT collaboratively and integrate future users.

### Structural hurdles for DATs’ implementation

The panels identified hurdles to implementing DAT in healthcare practice due to restrictive laws and lack of funding or technological infrastructure. A position paper on assistive technology policy also stated rigid legislative requirements can hamper innovations and investigations due to time and cost intensive processes. [61]. Astell and colleagues [16] raised questions of funding since common devices (e.g., smartphones) miss the classification as medical device and reimbursement by the public healthcare system. In Europe, in particular in Germany, strict regulations of the national German and European Medical Device Law [62] and General Data Protection Regulations [63] are relevant for DAT systems in healthcare. Medical device law aims to protect users from harm, but it also leads to high cost for certification [64]. In addition, companies need to provide detailed information about their technology and go through a time-consuming process which could be a barrier to certification, especially for start-ups. For more details on the certification process see Text Box 3. Our participants reflected this double role of legislation and regulatory requirements both as protection and hurdle for technology use which agrees with the results of Mac Lachlan and colleagues [61]. It was beyond the scope of the panels to discuss concrete changes in legislation or novel technologies that would meet regulatory requirements at affordable costs.

**Table:** 

Text Box 3: Additional information on the medical device certification process
In the European Economic Area, a medical device must be CE certified by the DQS med institute in order to be placed on the market [64]. Therefore, the DQS med institute requires a comprehensive application form with a detailed description on the technical documentation, the intended proposal, the risk classification, and the quality management system of the medical device [64]. Once the application has passed the initial review process, the DQS med will provide a cost estimate. Costs vary depending on, for example, the size of the company, the number of unannounced or announced audits or travel activities, with hourly rates for certification staff ranging from €300 to €600 per hour [64]. The certification process can take up to five years [64]. After successful certification, annual unannounced audits take place to ensure the quality of the medical device [64].

Participants identified the fact that the financing system for long-term care neglects investments in digitalization as a major hurdle. In nursing homes, investment costs that exceed the state subsidy are covered by contributions of the residents [65]. In Germany, the contribution amounts to 2,610 Euros on average [66]. On average, old-age pensioners receive 1,168 Euros [67] which is insufficient to cover the contribution in nursing homes. Therefore, nursing home managers may try to keep investment costs low at the expense of digitalization. As a result, even long standing technologies such as intercoms are missing in nursing homes, although participants identified them as very useful. A qualitative study from Sweden, focusing on digital healthcare communication revealed an improvement of care due to digital communication [68]. The participants recommended a mix of physical and digital communication to realize sufficient interaction with the patient [68].

Our participants regretted that the robotic seal PARO was not used in their nursing home. PARO’s fur is only antibacterial, but cannot be machine washed [36, 69]. As a result, the robot seal does not meet German hygiene standards for nursing homes [70]. Reviews found significant positive effects of PARO use on quality of life, affect, and social interaction [71, 72]. No studies from Germany could be found which supports the participant’s statement that PARO cannot be used in German nursing homes. This dilemma between possible benefits of PARO versus the potential risk of infection, highlights the value and need to involve users in the development of technologies to design marketable products.

In rural areas, the technological infrastructure is insufficient to support the use of web-based DATs. Our findings agree with the position papers from the first global research, innovation, and education on assistive technology (GREAT) on assistive technology policy and assistive technology products which identified rural areas as problem areas for access to DAT [18, 61]. Consistently, the position paper promotes different digital solutions not only for different users, but also for geographical locations [18]. In our view, a digitalization initiative by politicians, municipalities, future users, computer engineers, and healthcare managers is needed to bring healthcare up to the state of the art and provide patient-centered healthcare. In line with this, strengthening the digital competency of healthcare professionals and family caregivers should be taken into account from managers and politicians as well as the expansion of the technological infrastructure. Furthermore, interoperability between different systems and devices should be ensured in favor of devices’ compatibility and ease of use.

### Dementia-friendly communities instead of DAT

Besides DATs’ support, our participants saw wayfinding of people with dementia as general task for the society. They suggested that society as a whole (e.g., the baker) should be aware of disoriented people and be a contact person in case of need for help. Social responsibility, social inclusion and participation, remaining in one’s own living environment, sufficient technological infrastructure, and easy access to public transportation are conditions of dementia-friendly communities [73]. Dementia-friendly communities enable people with dementia to remain in their living environment by integrating, supporting, and promoting them [73]. In dementia-friendly communities, people with dementia, their families, organizations, and politicians cooperate to make the community aware of the social and occupational needs and rights of people with dementia by providing education, guidelines, and common activities [73]. However, in the literature of shaping dementia-friendly communities DATs play no or only a negative part [10, 73–76]. For instance, Shannon, Bail, and Neville [75] argue that online-based service applications such as self-checkout systems in libraries can confuse older people and prevent them from participating. For our participants, there seemed to be only one either-or perspective regarding the use of DAT as a navigational aid. In general, they were in favor of social interaction and dementia-friendly architecture and against the use of DAT. Our study revealed that the participants did not consider the possibility of combining both dimensions of support, technological and personal. However, combing physical and technological support might be useful as several reviews showed the benefit of DAT for people with dementia and their relatives [16, 77]. One illustrative example was provided by a participant with dementia from the online focus group. He proposed the use of Wi-Fi hotspots as an indicator of the life space zone. A DAT could assist the users by informing them when they leave a zone. In the event of disorientation, the DAT could provide a navigation aid with augmented reality, such as the live view in the Google Maps app. During navigation through the smartphone display, preset, well-known landmarks could be highlighted to assist the users in regaining their orientation. Dementia-friendly architecture and technical infrastructure could provide the hotspots as well as prominent landmarks. Therefore, we strongly recommend the integration of DAT as beneficial contributor to dementia-friendly communities.

### Strengths and limitations

A strength of our study lies in the multi-perspective view on ethical considerations and requirements for DAT for people with dementia. As people with dementia are often overlooked as stakeholders in AT [61], we provided a framework for discussion in which people with dementia and their relatives could participate on an equal footing and without stigma.

Another strength of our study is the transparent and participatory feedback process. By immediately taking notes of what was said, either on the paper tablecloth or on a digital whiteboard, we ensured congruence between what was said and what was documented. We sent the transcripts to the participants so they had the opportunity to comment. We received feedback from one participant. In the future, we will encourage our participants more intensively and collect feedback more actively, for example by telephone.

The results may be limited because the concept of mobility was very narrowly defined by our World Café participants (mobility as ability to walk independently). We have learned that for broad topics, the terms should be defined at the beginning and the moderator should more actively direct the discussion to other aspects, e.g. outdoor mobility. Although the World Café mainly covered the walking ability and indoor mobility, the results are transferable to other applications as well e.g., regarding the design of DAT and concrete use cases. To minimize this limitation, we discussed outdoor mobility separately in the online focus group as it is also an important aspect of mobility.

Participants discussed hurdles and problems regarding the implementation of DAT, but did not suggest opportunities and solutions. Typical limitations of focus group discussions arise due to insufficient speaking time per participant [35]. Focus groups can give an overview about relevant themes, but lack an in-depth or micro discussion [35]. The identified hurdles may be discussed in workshops to reveal solutions. Some discussed points, for example, political hurdles, focused on national law, are only applicable to the European Union.

In qualitative research, the moderator plays a central role in engaging interviewees to share their perspectives and feelings [35, 78]. It is inevitable that the moderator will influence the interview by bringing his or her character, skills, knowledge, and expectations into the moderation [78]. As we needed two moderators for the World Café discussion, moderator effects may have occurred [35]. In order to minimize this bias, both moderators discussed about difficulties and good practices in moderating a group discussion prior to the event.

Our results may also be limited by the time allowed for discussion. Our discussion took 30 to 40 min, but the participants could have gone beyond that. Following Patton [35], we recommend an extension to at least 60 min since this can lead to a deeper discussion and increase the space for follow-up questions. The time extension should be adapted to the concentration capacity of people with dementia.

Another limitation was the low recruitment rate. Unfortunately, three potential participants of the World Café discussion fell ill with COVID-19 at short notice, so we were unable to recruit additional participants. We also had to keep the number of participants low in order to reduce the risk of COVID-19 infection during the pandemic. Nevertheless, our number of participants is in line with Patton’s [35] recommendations regarding focus group discussions. Patton [35] recommends six to ten participants per group. In addition, other studies involving stakeholders and people with dementia have reported similar numbers of participants [79, 80]. Qualitative research does not claim to be representative. The added value of qualitative research lies in uncovering opinions, understanding processes and contexts, and in comparing and discussing different points of view [35]. Our study was able to achieve these aims despite the small number of participants.

## Conclusions

Our investigation gave in-depth insights into ethical concerns and requirements for DAT supporting people with dementia from multiple perspectives. Participants discussed autonomy, independence, decision making capacity, and decision-making strategies, such as nudging or incentives, as well as structural, political, and financial hurdles in implementing DAT in healthcare and supporting mobility. The design requirements were individual, but all participants agreed that DAT must adapt to the user not the user to the DAT. In summary, our study provides concrete ethical and technological requirements for the development of DAT.

Our participants valued interpersonal interaction and social responsibility very highly. They highlighted the importance of interpersonal interaction and worried that DAT could reduce human interaction. Therefore, future studies should examine how the implementation of AT influences the interaction of people with dementia, their caregivers, and their environment. Identifying and talking about these concerns with the future users is necessary to gain acceptance for DAT. Designers and researchers in the field of DAT should implement participatory design methods such as user-centered design to develop marketable and beneficial DATs incorporating users’ needs and values. In the same line, concepts for dementia-friendly communities integrating DAT solutions must be developed.

### Supplementary Information

Supplementary Material 1- Moderation table 1.

Supplementary Material 2- Moderation table 2.

Supplementary Material 3- Online focus group guideline.

## Data Availability

The protocols generated during and/or analyzed during the current study are available in English language from the corresponding author on reasonable request.
