# Supplementary material for: Ethics, design, and implementation criteria of digital assistive technologies for people with dementia from a multiple stakeholder perspective: a qualitative study
Source: BMC Med Ethics. 2024 Jul 27;25:84. doi: 10.1186/s12910-024-01080-6 (PMC11282641; doi:10.1186/s12910-024-01080-6)
Supplement: Supplementary file 1 — Supplementary Material 1- Moderation table 1. [file 12910_2024_1080_MOESM1_ESM.docx]

# Moderation Table 1

**Agenda:**- Greeting 5 min
- Introduction of the participants 5 min
- Presenting the question to be discussed 30 min
- Summary of the results 5 min

# **Question: „Which needs should AT address to support people with dementia and healthcare in dementia?”**

**Supplementary questions:**

- During the short impulse presentations you have gained an insight into the possibilities of technical assistance systems. In which area(s) do you think digital solutions should be used?
- With which goal should digital solutions be used?
- To whom should this support be addressed?
- What opportunities and risks do you see for support with digital applications?
- Who should be involved in projects to develop digital solutions? Would you participate in such a project yourself?
