# Supplementary material for: Ethics, design, and implementation criteria of digital assistive technologies for people with dementia from a multiple stakeholder perspective: a qualitative study
Source: BMC Med Ethics. 2024 Jul 27;25:84. doi: 10.1186/s12910-024-01080-6 (PMC11282641; doi:10.1186/s12910-024-01080-6)
Supplement: Supplementary file 2 — Supplementary Material 2- Moderation table 2. [file 12910_2024_1080_MOESM2_ESM.docx]

# Moderation Table 2

**Agenda:**- Greeting 5 min
- Introduction of the participants 5 min
- Presenting the question to be discussed 30 min
- Summary of the results 5 min

# **Question: „Which application fields, needs, and benefits exist regarding AT supporting mobility? What are limitations and hurdles?”**

**Supplementary questions:**

- Would you be concerned about your data? (Issues with data protection?)
- Which mobile assistants are already used?
- What should a mobile assistant be able to do? (e.g., prevention of running away, fall prevention).
