# Supplementary material for: Ethics, design, and implementation criteria of digital assistive technologies for people with dementia from a multiple stakeholder perspective: a qualitative study
Source: BMC Med Ethics. 2024 Jul 27;25:84. doi: 10.1186/s12910-024-01080-6 (PMC11282641; doi:10.1186/s12910-024-01080-6)
Supplement: Supplementary file 3 — Supplementary Material 3- Online focus group guideline. [file 12910_2024_1080_MOESM3_ESM.pdf]

Guideline for the online focus group

Agenda:
- Greeting 5 min
- Warm-Up and introduction of the participants 10 min
- Presenting the question to be discussed 30 min
- Summary of the results 10 min
- Farewell and goodbye 5 min

Warm-up and introduction of the participants

„Digital assistive systems for people with dementia, caregiving relatives and healthcare professionals“

What is your background and what symbol do you associate with mobility?

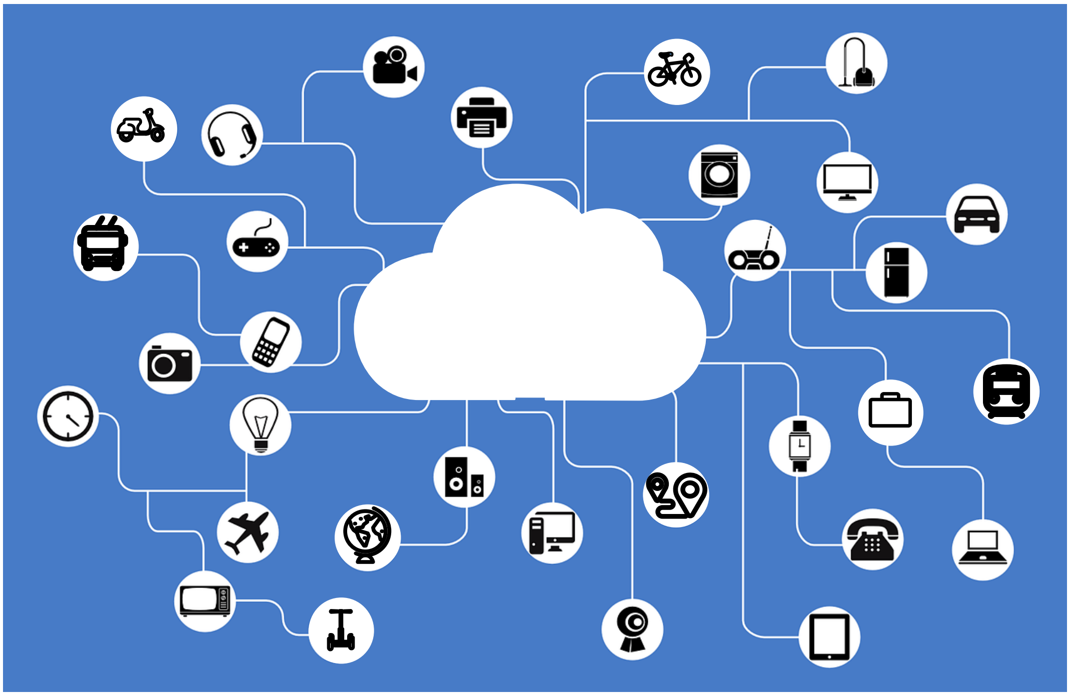

Questions

1. How can AT support outdoor mobility of people with dementia?

2. Which differences exist regarding outdoor mobility needs between urban and rural areas?

3. Which chances and risks exist? Which general conditions must be fulfilled?

Supplementary question

Who do you think should be responsible for shutting down a system?
